# Complementary and Alternative Medicine in Midwifery

**Published:** 2014-06-15

**Authors:** Masoumeh Abedzadeh Kalahroudi

**Affiliations:** 1 Trauma Nursing Research Center, Kashan University of Medical Science, Kashan, IR Iran

**Keywords:** Complementary Medicine, Alternative Medicine, Holistic Care

The World Health Organization defines “complementary and alternative medicine” (CAM) as a “broad set of health care practices that are not part of that country’s own tradition and are not integrated into the dominant health care system” (1). Complementary therapy is usually used in combination with current treatments, and alternative treatments are used instead of current treatments. Commonly utilized types of CAM include acupuncture, aromatherapy, herbal and homeopathic medicines, meditation, movement therapies, chiropractic and osteopathic manipulation and so on (2). An overview of the recent research around the world showed that in developing country 5-74.8% of people use these therapeutic methods especially for chronic diseases (3). In Iran, some of these methods such as herbal medicine have been used for thousands of years and are rapidly expanding. There is no clear estimation of CAM users in Iran but studies showed that 62.5% of people in Isfahan city and 66.3% in Tehran city use CAM therapy (4, 5). Women use CAM more than men. Many women prefer to use CAM during pregnancy because of fewer side effects compared to chemical drugs. Studies have shown that 73% of pregnant mothers in Australia (6) and 83.7% in Mashhad city used CAM during pregnancy (7). Today, around the worldwide, midwives use complementary therapies in their profession more than the other medical practitioners. A literature review estimated that between 65% - 100% of midwives have used one or more complementary therapies. Common types of CAM that are recommended by midwives were: massage therapy, herbal medicines, relaxation techniques, nutritional supplements, aromatherapy, homeopathy and acupuncture (8). However, one survey in Iran found that CAM methods are prescribed by 37.3% of obstetricians with the most common methods being acupressure, massage and herbal therapy (9). Another study also showed that 44% of midwifery students had recommended CAM therapies to others (10). These methods can be used for treatment of nausea and vomiting, back pain, anxiety, postpartum depression, anemia, striae gravidarum, insomnia, hemorrhoid, vaginal infection, mal-presentation, augmentation and induction of labor, perineal care, retained placenta and lactation problems (8, 11, 12). Since midwives are care providers to women during puberty and reproductive periods, especially at pregnancy and also during menopause and post-menopausal periods, therefore use of complementary and alternative therapies gives an opportunity to midwives for providing holistic care and enables them to respond to the community and women’s needs. Hence, all midwives who are interested in/or are practicing CAM for their clients should be empowered in this field through participating in educational programs to acquire the necessary skills. In midwifery training programs, evidence based CAM therapy should be included. Midwives not only need to know the strengths and limitations of CAM methods, but also they should be able to talk to women about the effectiveness and possible risks of these procedures. Given the widespread use of CAM in the midwifery field, it is necessary for medical organizations to prepare relevant guidelines for using these medicines in midwifery practice, especially for maternity care. It seems that further research to evaluate the prevalence, safety, efﬁcacy and economic benefits of these methods are required.

## References

[1] World Health Organisation. (2013.). Traditional Medicines: Definition..

[2] (2014.). National Center for Complementary and Alternative Medicine..

[3] Frass M, Strassl RP, Friehs H, Mullner M, Kundi M, Kaye AD (2012). Use and acceptance of complementary and alternative medicine among the general population and medical personnel: a systematic review.. Ochsner J..

[4] Banihashemi Tehrani SA, Asgharifard H, Haghdoust AA, Barghamadi M, Mohammad Hosseini N (2008). [The use of Complementary/Alternative Medicine among the general population in Tehran, Iran].. Payesh..

[5] Yekta Z, Zamani A, Mehdizade M, Farajzadegan Z (2007). Pattern of complementary and alternative medicine use in urban population.. J Res Health Sci..

[6] Skouteris H, Wertheim EH, Rallis S, Paxton SJ, Kelly L, Milgrom J (2008). Use of complementary and alternative medicines by a sample of Australian women during pregnancy.. Aust N Z J Obstet Gynaecol..

[7] Khadivzadeh T, Ghabel M (2012). Complementary and alternative medicine use in pregnancy in Mashhad, Iran, 2007-8.. Iran J Nurs Midwifery Res..

[8] Hall HG, McKenna LG, Griffiths DL (2012). Midwives' support for Complementary and Alternative Medicine: a literature review.. Women Birth..

[9] Fahimi F, Hrgovic I, El-Safadi S, Münstedt K (2011). Complementary and alternative medicine in obstetrics: a survey from Iran.. Arch Gynecol Obstet..

[10] Adib-Hajbaghery M, Hoseinian M (2014). Knowledge, attitude and practice toward complementary and traditional medicine among Kashan health care staff, 2012.. Complement Ther Med..

[11] Saberi F, Sadat Z, Abedzadeh-Kalahroudi M, Taebi M (2013). Acupressure and ginger to relieve nausea and vomiting in pregnancy: a randomized study.. Iran Red Crescent Med J..

[12] Saberi F, Sadat Z, Abedzadeh-Kalahroudi M, Taebi M (2014). Effect of Ginger on Relieving Nausea and Vomiting in Pregnancy: A Randomized, Placebo-Controlled Trial.. Nurs Midwifery Stud..

